# Disseminated *Nocardia nova* in a child with relapsed acute lymphoblastic leukemia: a case report

**DOI:** 10.1186/s12879-023-08895-5

**Published:** 2024-02-01

**Authors:** Victor Arahirwa, Sahal Thahir, Lauren Hernandez, Zachary Inskeep Willis

**Affiliations:** University of North Carolina School of Medicine, Chapel Hill, USA

**Keywords:** Nocardiosis, Bacteremia, Acute lymphoblastic leukemia

## Abstract

**Background:**

Nocardiosis is a rare infection that typically results from inhalation of or inoculation with *Nocardia* organisms. It may cause invasive disease in immunocompromised patients. This case describes nocardiosis with bacteremia and pulmonary involvement in a child with a hematologic malignancy.

**Case presentation:**

A boy with testicular relapsed acute lymphoblastic leukemia with marrow involvement presented with sudden onset of fever, body aches, headaches, chills, and moderate respiratory distress during continuation 2 chemotherapy. Radiographic imaging demonstrated consolidation and ground glass opacities in bilateral lower lungs. Central line blood cultures grew *Nocardia nova* complex, prompting removal of the central line and initiation of triple therapy with imipenem-cilastatin, linezolid, and trimethoprim-sulfamethoxazole with rapid improvement of symptoms. Antibiotic susceptibilities showed a multidrug-susceptible isolate. The patient is anticipated to remain on trimethoprim-sulfamethoxazole for at least 12 months.

**Conclusions:**

In an immunocompromised child, blood cultures, chest imaging, and head imaging can aid in the diagnosis of disseminated nocardiosis. Long-term antibiotic therapy is necessary, guided by the organism and simplified with the results of antimicrobial susceptibility testing.

## Background

Nocardiosis is an infection caused by *Nocardia*, a genus of partially acid-fast, aerobic, gram-positive actinomycetes that are found ubiquitously in soil, fresh water, and marine water [1]. Infections typically result from inhalation of contaminated particles, but can also occur by direct skin penetration [1, 2]. Nocardiosis is generally considered an opportunistic infection, with most cases occurring in immunocompromised patients [3]. Nocardiosis most frequently presents with pulmonary disease, followed by disseminated disease, and central nervous system (CNS) disease [3, 4]. About one-third of patients will have disseminated disease at presentation, possibly due to hematogenous spread from pulmonary or cutaneous sites of inoculation [5]. However, isolation of *Nocardia* isolates from blood culture is rare, even in immunocompromised patients with underlying malignancies [1, 6]. *Nocardia* bacteremia is most frequently identified in immunocompromised patients and those with intravascular devices, and represents a serious infection with high overall mortality [3]. We report here the case of an immunocompromised child with *Nocardia* bacteremia with pulmonary involvement.

## Case presentation

A 6-year-old boy with testicular relapsed B-cell acute lymphoblastic leukemia (B-ALL) with marrow involvement presented with fever, body aches, headaches, and chills. He was in his usual state of health until noon that day, when he started to feel ill. He felt warm to the touch with an oral temperature of 38.9 C (102 F). He had no other symptoms and no known sick contacts. His only home medication was trimethoprim-sulfamethoxazole (TMP-SMX), which he took on Saturdays and Sundays for *Pneumocystis jirovecii* prophylaxis. His last chemotherapy treatment was one week prior and consisted of intravenous (IV) etoposide and IV cyclophosphamide.

Nine months prior to the patient’s presentation, in August 2022, he was noted to have an enlarged, firm right testicle during routine follow-up. A testicular biopsy, bone marrow biopsy, and lumbar puncture confirmed testicular relapsed B-ALL with marrow involvement. Following this diagnosis, the patient began chemotherapy, radiation therapy, and immunotherapy with blinatumomab. He had been intermittently neutropenic between October and December 2022.

In February 2023, he began an eight-week cycle of chemotherapy, known as continuation 2, which consisted of oral mercaptopurine, oral methotrexate, oral thioguanine, IV vincristine, IV etoposide, IV cyclophosphamide, and intrathecal methotrexate. He had just completed this phase of treatment and was due to start maintenance therapy with oral dexamethasone, oral methotrexate, oral mercaptopurine, IV vincristine, and intrathecal methotrexate when he became ill. Thus, the start of maintenance therapy was deferred to the following week, when the patient was more clinically stable.

At initial presentation (day 0), the patient was tachycardic, non-toxic, and well-hydrated. He had a tunneled central venous catheter (CVC) with no surrounding erythema. Labs were significant for white blood cell count 2,800/μL, absolute neutrophil count 2,100/mm^3^ (increased from 600/mm^3^ the week prior and 300/mm^3^ one month prior), hemoglobin 9.1 g/dL, and platelets 60,000/μL. He received ceftriaxone and a normal saline bolus. Respiratory pathogen panel was negative. He was admitted to the pediatric hematology-oncology service for observation.

On day 1, the patient became septic with tachycardia, hypotension, and fever to 39.5 C (103.1 F), requiring three normal saline boluses of 20 mL/kg each. C-reactive protein level was 148 mg/L. Antibiotics were broadened to vancomycin and cefepime, given his ill appearance and immunocompromised state. He was ultimately transferred to the pediatric intensive care unit after developing altered mental status. A repeat chest X-ray showed new diffuse patchy airspace opacities and focal right lower lung confluent opacities, in the setting of a new oxygen requirement. The patient’s mental status rapidly improved and was thought to be behavioral in nature. He was transferred back to the floor on day 2.

**Fig. 1:**
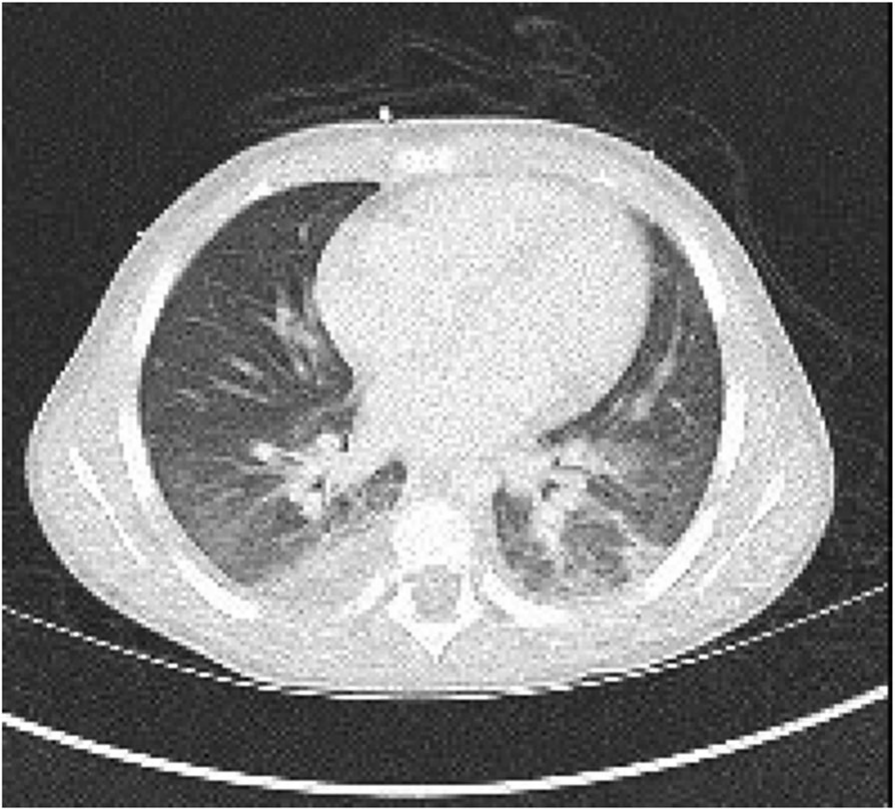
Lung bases captured by abdominal computed tomography on day 2. Shown in the lungs are bilateral lower lobe consolidation and interdigitating ground glass opacities

**Fig. 2:**
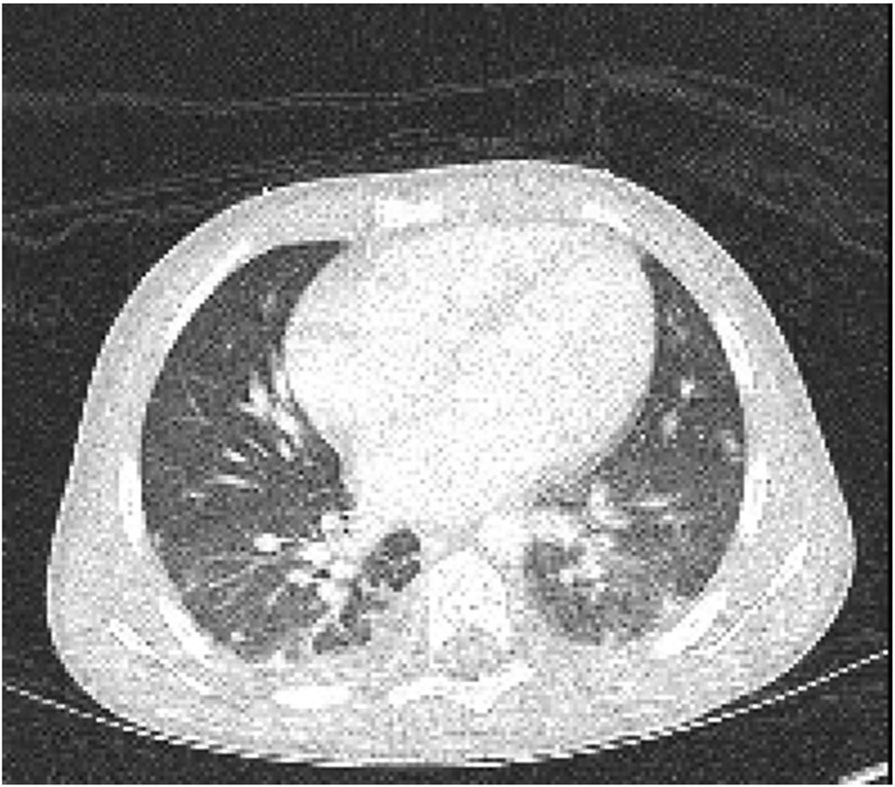
Lung bases captured by chest computed tomography on day 6. Shown in the lungs are numerous peripheral ground glass opacities bilaterally with mild central bronchial wall thickening

On day 2, CT scan of the abdomen and pelvis was obtained due to abdominal pain and demonstrated bilateral lower lobe consolidation and ground glass opacities concerning for pneumonia, small bilateral pleural effusions, and splenomegaly (Fig. 1). His fever persisted, and he was noted to have severe neutropenia, with an absolute neutrophil count of 500/μL. Blood cultures collected from each lumen of the patient’s tunneled double-lumen CVC on day 0 began to grow gram-positive rods after 36 h. On day 3, the organisms were identified as *Nocardia nova* complex. He had multiple subsequent central and peripheral blood cultures on days 1 and 3, only one of which, a CVC culture collected on day 3, also grew *N. nova*. An isolate was sent to Mayo Clinic Laboratories (Rochester, MN) for antimicrobial susceptibilities. Following identification of *N. nova*, vancomycin and cefepime were discontinued, and the patient was started on imipenem-cilastatin, TMP-SMX, and linezolid. Over the next day, fever and oxygen requirement resolved. The CVC was removed on day 4 after being in place for 256 days. Due to the episode of altered mental status, CT head, lumbar puncture, and brain MRI were performed, with unremarkable results. On day 6, CT chest revealed mild central bronchial wall thickening and numerous peripheral nodular and ground glass opacities without evidence of abscess (Fig. 2). Because he had already improved without antifungal therapy, these findings were attributed to nocardiosis; dedicated evaluation for invasive fungal infection was not pursued. On day 7, following blood culture clearance, a peripherally inserted central catheter was placed for continuation of antibiotics and chemotherapy.

The patient began maintenance chemotherapy on day 8, consisting of intrathecal methotrexate, IV vincristine, oral prednisone, and oral mercaptopurine. He was discharged on day 11 with imipenem-cilastatin, TMP-SMX, and linezolid, pending antimicrobial susceptibilities. Susceptibility testing was reported on day 12, showing susceptibility to ceftriaxone, imipenem, clarithromycin, amikacin, TMP-SMX, and linezolid (Table 1). Imipenem-cilastatin was discontinued at that time. The patient was unable to tolerate large doses of TMP-SMX suspension and unable to swallow pills, so linezolid was continued and azithromycin was added. Six weeks after discharge, linezolid and azithromycin were discontinued in favor of TMP-SMX monotherapy at a dose of 800-160 mg twice daily (10.3 mg of trimethoprim per kilogram per day). Since TMP-SMX and methotrexate are both anti-folate drugs, and due to the patient’s recent pancytopenia, the dose of oral methotrexate was reduced by 42%. Follow-up chest X-ray 19 days after discharge showed interval improvement in bilateral patchy perihilar opacities. At his outpatient visits, he was well-appearing and hemodynamically stable, apart from an episode of COVID-19 infection in August 2023, treated with remdesivir. He is set to remain on TMP-SMX for a prolonged course, with periodic chest CT to evaluate response.

**Table 1:** Antimicrobial susceptibility of the *Nocardia nova* complex isolate (μg/mL)^a,b,c^

Antibiotic	MIC (μg /mL)	Interpretation
Amoxicillin/clavulanic acid	32/16	R
Ceftriaxone	8	S
Imipenem	1	S
Ciprofloxacin	16	R
Moxifloxacin	4	R
Clarithromycin	0.12	S
Amikacin	≤ 0.5	S
Tobramycin	> 64	R
Doxycycline	16	R
Minocycline	4	I
Trimethoprim/sulfamethoxazole	0.25/4.75	S
Linezolid	2	S

*MIC* minimum inhibitory concentration, *S* susceptible, *I* intermediate, *R* resistant

^a^Ciprofloxacin: Ciprofloxacin and levofloxacin are interchangeable, but both are less active in vitro than moxifloxacin

^b^Clarithromycin: Class representative for all macrolides except erythromycin

^c^Antimicrobial susceptibility testing was performed by using the microdilution method according to the Clinical and Laboratory Standards Institute standard M24-A2 (CLSI, 2011)

## Discussion

We describe a rare case of *Nocardia* bacteremia with pulmonary involvement in an immunocompromised child with B-ALL. Imaging revealed nodular lung densities. This patient’s fevers prompted physicians to obtain blood cultures, which grew *N. nova* complex. Nocardiosis is often a challenging diagnosis to make, as bacteremia is not common, and the pulmonary imaging findings are very similar to those in invasive fungal infection. Typical empiric coverage in this scenario would likely not include optimal coverage of nocardiosis, and definitive therapy requires prolonged antibiotic courses. Like invasive fungal infections in this population, there is risk of dissemination to CNS. Therefore, awareness of this condition and prompt diagnosis is critical.

Aerobic actinomycetes are found in a broad geographic distribution, and pathogenic species can be found in house dust, beach sand, garden soil, and swimming pools [1]. Inoculation is most commonly via inhalation or penetrating skin trauma. Nosocomial nocardial infection has been reported only rarely. In our patient, the source of the infection is unclear; with the prominent pulmonary findings, inhalation of contaminated particles in the environment seems most likely. There is a high proportion of male patients [3], immunosuppressed patients, in particular due to corticosteroid use [3, 4], solid organ or hematopoietic stem cell transplantation [7], and hematologic malignancy [3, 4]; and patients with intravascular devices [3, 6, 8] in retrospective case series and collections of case reports of *Nocardia* infection. In adults, especially in the setting of hematologic malignancy, cases of apparently primary catheter-related bloodstream infection (CRBSI) without dissemination have been reported [8].

*N. nova* complex was the most commonly identified causative species in a prior series of *Nocardia* CRBSI [8], although this complex has recently been identified in about 20% of clinically significant isolates [9]. In laboratory models, *Nocardia* spp. have the ability to adhere to CVCs through biofilm formation on the surface of catheters [8]. On this basis, we proceeded with immediate CVC removal in this case. However, trimethoprim- and minocycline-based lock solutions have potent in vitro activity against biofilm formation [8], and, in one report, a child with central line-associated nocardiosis due to *N. otitidis caviarum* was successfully treated without catheter removal [10]. Whether CVC removal should be pursued in all cases of CVC-associated nocardiosis remains uncertain in the absence of controlled comparisons of catheter removal versus salvage strategies.

*Nocardia* infection is rare in pediatric patients, presenting historically as lung disease in immunocompromised hosts [11–13]. In 1957, Ballenger and Goldring reported a fatal case of nocardiosis, clinically simulating tuberculosis, in a 45-month-old patient [11]. The patient had a chronic pulmonary disease characterized by persistent cough, intermittent fever, hepatosplenomegaly, generalized lymphadenopathy, anemia, growth failure, and pulmonary insufficiency. Exudate from the lungs, liver, spleen, and thyroid at the time of autopsy yielded *N. asteroides*. The patient showed an apparent response to sulfonamides several times during the course of his illness. In the authors' review of twelve pediatric cases, six children had pulmonary involvement and three children had CNS involvement. Nine of the 12 children died.

In 1963, nocardiosis was reported in the case of a 7-year-old patient who presented with fever, cough, malaise, thoracic pain, and polymorphonuclear leukocytosis. He had an acute, fulminating, widely disseminated form of the disease, including meningitis. Bronchial aspirate culture yielded *N. asteroides*, and he was started on sulfadiazine after failing to respond to prior antibiotics. He died shortly thereafter [12]. In 1975, Idriss et al. described three cases of *N. asteroides* infection in adolescents, who all improved steadily with sulfonamide therapy. At that time, 35 pediatric cases had been reported in the literature. Twenty-eight cases were ascribed to *N. asteroides* or described only as *Nocardia* species, five cases were caused by *N. brasiliensis*, and six cases were in patients with chronic granulomatous disease [13].

In a report of *Nocardia* infection in ten children between 1975 and 1980, three immunocompromised children developed fatal *N. asteroides* infection. Each presented with pneumonia, and one had disseminated disease. One child developed shunt-associated *N. asteroides* ventriculitis. *N. brasiliensis* was isolated from six immunocompetent children, five of whom had localized, uncomplicated, cutaneous infections [14]. Nocardiosis has also been reported in children with cystic fibrosis [15] and renal transplantation [16].

A few cases of invasive nocardiosis have been reported in immunocompetent children [17–19]. In a previously healthy child initially admitted with an abdominal mass, abdominal pain, and progressive altered mental status, laboratory and imaging findings revealed lymphocytic meningitis and disseminated abscesses in the brain and a large number of cystic lesions of the kidney. The patient received broad-spectrum antibiotics, as well as antituberculous and antifungal therapies, and died 6 days after admission. A multidrug-resistant isolate of the *N. elegans/aobensis/africana* complex was cultured from a renal biopsy and cerebrospinal fluid [17]. Nocardiosis with sinusitis [18] and CNS granulomatous inflammation [19] have also been reported in immunocompetent children. In these cases, the authors reported initial misdiagnosis, presumably led by transient improvement under a broad-spectrum antibiotic.

In the United States, there has been one reported case of *N. nova* CVC infection complicated by secondary dissemination to the lungs in a child with ALL. He presented with fevers and chest CT showing nodular lung densities. Following CVC removal, three successive daily blood cultures obtained from the CVC revealed *N. nova*. He was treated with ceftriaxone and amikacin for 4 weeks, amoxicillin and clarithromycin for an additional 4 weeks, and subsequently clarithromycin for 6 months, leading to complete resolution of lung lesions [20].

In adult patients, one systematic review described improved outcomes in patients with *Nocardia* bacteremia associated with intravascular devices, with a low 30-day mortality observed in this group (8%) [3]. In this review, blood cultures were an important diagnostic method, representing the only positive microbiological specimen in 38% of cases. In our case, the presence of negative blood cultures on day 1 after positive cultures may have been due to insufficient blood culture sampling or inadequate duration of incubation, with up to 14 days required to identify visible colonies of *Nocardia* on solid media [3].

TMP-SMX remains the treatment of choice for most *Nocardia* infections [1, 2]. In addition, TMP-SMX is widely used to prevent *Pneumocystis jirovecii* infection in children with hematologic malignancies [21]. However, the dose of TMP-SMX commonly used for *Pneumocystis* prophylaxis is not protective against *Nocardia* [22]. A combined antibiotic therapy (generally the addition of amikacin and carbapenem) is preferred for immunosuppressed patients with local disease or for disseminated infections when the diagnosis is suspected [4, 7].

The antimicrobial susceptibility pattern of *Nocardia* isolates differs among species [23]. Therefore, the antibiotic regimen should be guided by the involved species and then simplified based on the results of susceptibility testing [7]. Our patient’s isolate was susceptible to TMP-SMX, imipenem-cilastatin, and linezolid. However, as reported in other studies, *N. nova* complex was characterized by its susceptibility to clarithromycin and resistance to amoxicillin and clavulanic acid [8]. This distinctive characteristic of *N. nova* complex is associated with the presence of membrane-bound penicillinase inducible by clavulanic acid [24].

Optimal duration of therapy for *Nocardia* infections is uncertain, but long-term therapy is favored due to the potential for relapse [1]. Immunocompetent patients with pulmonary or systemic nocardiosis (excluding CNS involvement) should be treated for a minimum of 6–12 months. Our patient will likely require at least 12 months of therapy, as is the minimum recommended duration of therapy in immunosuppressed patients [1, 2].

## Conclusion

In immunosuppressed children, including those receiving treatment for malignancy, blood cultures, chest imaging, and head imaging should be obtained, as disseminated nocardiosis may occur. Prompt empiric antibiotic therapy should be initiated, and then antibiotic selection should be tailored based on antimicrobial drug susceptibility. Immunosuppressed children should remain on antibiotics for at least 12 months.

## Data Availability

All data generated or analyzed during this study are included in this published article.

## Abbreviations

CNS: Central nervous system
B-ALL: B-cell acute lymphoblastic leukemia
TMP-SMX: Trimethoprim-sulfamethoxazole
IV: Intravenous
CVC: Central venous catheter
CT: Computed tomography
MRI: Magnetic resonance imaging
CRBSI: Catheter-related bloodstream infection
BAL: Bronchoalveolar lavage
